# Comparative genome analysis reveals the molecular basis of nicotine degradation and survival capacities of *Arthrobacter*

**DOI:** 10.1038/srep08642

**Published:** 2015-02-27

**Authors:** Yuxiang Yao, Hongzhi Tang, Fei Su, Ping Xu

**Affiliations:** 1 State Key Laboratory of Microbial Metabolism, and School of Life Sciences & Biotechnology, Shanghai Jiao Tong University, Shanghai 200240, People's Republic of China; 2 Joint International Research Laboratory of Metabolic & Developmental Sciences, Shanghai Jiao Tong University, Shanghai 200240, People's Republic of China

## Abstract

*Arthrobacter* is one of the most prevalent genera of nicotine-degrading bacteria; however, studies of nicotine degradation in *Arthrobacter* species remain at the plasmid level (plasmid pAO1). Here, we report the bioinformatic analysis of a nicotine-degrading *Arthrobacter aurescens* M2012083, and show that the *moeB* and *mogA* genes that are essential for nicotine degradation in *Arthrobacter* are absent from plasmid pAO1. Homologues of all the nicotine degradation-related genes of plasmid pAO1 were found to be located on a 68,622-bp DNA segment (*nic* segment-1) in the M2012083 genome, showing 98.1% nucleotide acid sequence identity to the 69,252-bp *nic* segment of plasmid pAO1. However, the rest sequence of plasmid pAO1 other than the *nic* segment shows no significant similarity to the genome sequence of strain M2012083. Taken together, our data suggest that the nicotine degradation-related genes of strain M2012083 are located on the chromosome or a plasmid other than pAO1. Based on the genomic sequence comparison of strain M2012083 and six other *Arthrobacter* strains, we have identified 17 σ^70^ transcription factors reported to be involved in stress responses and 109 genes involved in environmental adaptability of strain M2012083. These results reveal the molecular basis of nicotine degradation and survival capacities of *Arthrobacter* species.

A*rthrobacter* species, which were first isolated from soil in 1889^1^, are high GC-content bacteria, and typically appear as gram-negative rods in early cultivation stages, but as gram-positive cocci in later stages^2^. Strains of these species are among the most common aerobic culturable bacteria in soil and are thought to act as the predominant decomposers of organic matter^3^. *Arthrobacter* species have been reported to be able to degrade environmental pollutants such as nitroglycerin, many benzene derivatives, polycyclic aromatic compounds, haloalcohols, haloalkanes, *N*-heterocyclic compounds, insecticides, and herbicides^4^,^5^,^6^,^7^,^8^,^9^,^10^,^11^,^12^,^13^,^14^,^15^,^16^,^17^,^18^,^19^. They have been found in extreme environments such as arctic ice, the deep subsurface, and radioactive environments; in addition, chemically contaminated and heavy metal-contaminated sites seem to be rich in these bacteria^20^,^21^,^22^,^23^,^24^. The ubiquity of *Arthrobacter* species is probably due to their tolerance to various stresses such as long-term starvation, desiccation, oxidative stress, temperature shifts, osmotic pressure changes, ionizing radiation, excess heavy metal ions, and toxic chemicals^22^,^23^,^24^,^25^,^26^,^27^,^28^,^29^. Their remarkable survival abilities contribute to the importance of *Arthrobacter* species in pollutant degradation in complex and volatile environments^27^. Therefore, in order to reveal the application potential of a pollutant-degrading bacterium in environmental remediation, it is necessary to understand not only the genes involved in pollutant degradation but also the genes involved in environmental survivability.

Because of the widespread presence and high toxicity of *N*-heterocyclic pollutants, their degradation has received great attention^30^. Some *N*-heterocyclic pollutant-degrading *Arthrobacter* species such as the *s*-triazine-degrading strain *A. aurescens* TC1 (TC1) and quinaldine-degrading strain *Arthrobacter* *nitroguajacolicus* Rue61a (Rue61a) have been isolated from atrazine-containing soil and the sewage sludge of a coal tar refining factory, respectively^18^,^31^,^32^. Furthermore, their complete genomes have been sequenced, and the molecular bases of their metabolic and survival potentials have been thoroughly analyzed^27^,^33^.

The complete genome sequences of four other *Arthrobacter* species including three soil strains, *Arthrobacter* sp. FB24 (FB24), *Arthrobacter chlorophenolicus* A6 (A6), and *Arthrobacter phenanthrenivorans* Sphe3 (Sphe3), and a food-processing strain, *Arthrobacter arilaitensis* Re117 (Re117), were reported. Strains FB24, A6 and Sphe3 were isolated by their ability to degrade dimethylbenzene, 4-chlorophenol, and phenanthrene, respectively. Genome analyses revealed many metabolic and stress-related genes in these environmental strains, which reflected their pollutant-degrading abilities and niche specializations. Strain Re117 was isolated from the surface of Reblochon cheese; its genetic adaptation to the habitat via genes such as those involved in iron acquisition, salt tolerance, and catabolism of fatty acids, amino acids, and lactic acid, which are major carbon substrates present at the cheese surface, was also revealed by genome sequence analysis^32^,^34^,^35^,^36^.

Although nicotine is a typical *N*-heterocyclic pollutant and *Arthrobacter* is one of the most prevalent genera of nicotine-degrading bacteria, studies of nicotine degradation by *Arthrobacter* species remain at the plasmid level, while the complete genome of another prevalent bacterium, *Pseudomonas putida* S16, has been sequenced and extensively studied^37^,^38^,^39^,^40^,^41^,^42^,^43^. A catabolic plasmid of 165-kb isolated from *Arthrobacter nicotinovorans* (formerly classified as *Arthrobacter oxydans*) was sequenced, and almost all the genes involved in nicotine degradation by the bacterium were shown to locate on it^37^,^38^,^44^. Two related genes that were absent from the plasmid are required for molybdenum cofactor (MoCo) biosynthesis; one encodes MoeB, which is essential for ATP-dependent activation of the MoaD subunit of molybdopterin (MPT) synthase; the other encodes a MoaB/MogA homolog, which is probably required for MPT-adenylate formation^38^,^45^. These genes must locate on the chromosome, and genome sequence analysis will help us to establish this. Furthermore, the survival capacity of nicotine-degrading *Arthrobacter* strains, which is very important to their application potential, is still unknown. Therefore, it is necessary to obtain and analyze the genome sequence of a nicotine-degrading *Arthrobacter* strain in order to uncover the molecular basis of its survival capacity.

Many catabolic plasmids are thought to take part in the spread of catabolic traits by horizontal gene transfer between bacteria^46^,^47^,^48^. Recently, Mihasan and Brandsch proposed that the nicotine-degrading genes of *A. nicotinovorans* were possibly obtained via plasmid pAO1 or via a precursor plasmid from the chromosome of a strain that was probably related to *Rhodococcus opacus* or another *Arthrobacter* species. However, *Arthrobacter* species harboring nicotine-degrading genes on the chromosome have not yet been reported^39^.

Recently, we isolated a new nicotine-degrading strain, *Arthrobacter* sp. M2012083 (M2012083), from tobacco waste, and obtained and published its genome sequence, but without further analysis^49^. In the present study, we classified this strain as *Arthrobacter aurescen* by physiological and biochemical identification, 16S rDNA phylogenetic analysis, and genomic analysis; we compared the genomic sequence of strain M2012083 with the six available *Arthrobacter* complete genomes and the sequence of nicotine-degrading plasmid pAO1, in order to provide a better understanding of the genetic basis of the nicotine-degradation and survival capacities of this genus.

## Results

### Classification of strain M2012083

**Figure 1:**
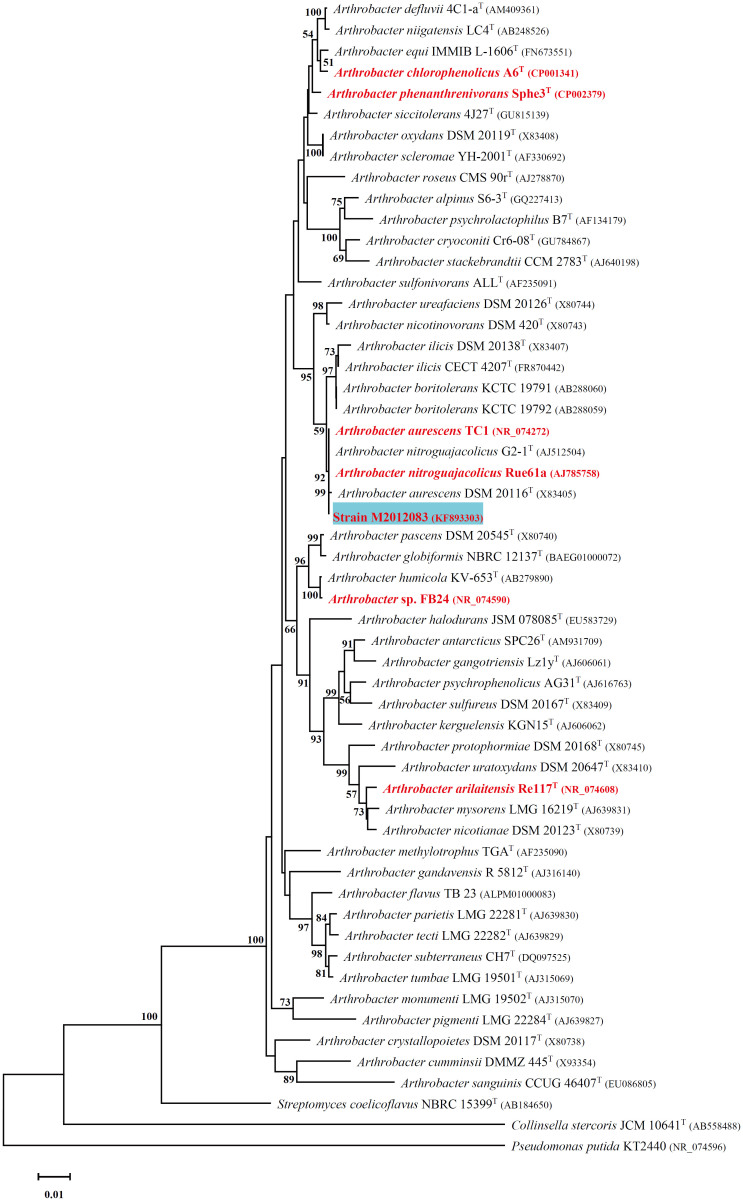
16S rDNA phylogenetic analysis of *Arthrobacter* strains. The evolutionary history was inferred using the Neighbor-Joining method^52^. The optimal tree with the sum of branch length = 0.82 is shown. The percentage of replicate trees in which the associated taxa clustered together in the bootstrap test (1000 replicates) are shown next to the branches^72^. The tree is drawn to scale, with branch lengths in the same units as those of the evolutionary distances used to infer the phylogenetic tree. The evolutionary distances were computed using the Neighbour-Joining (NJ) method^70^ and are in the units of the number of base substitutions per site. The analysis involved 55 nucleotide sequences. All positions containing gaps and missing data were eliminated. There were a total of 1,327 positions in the final DataSet. Evolutionary analyses were conducted in MEGA5^51^. Only bootstrap values greater than 50% are shown. The strains used in genome comparison are shown in red, the type strains are indicated by superscript T and the strain M2012083 is highlighted in blue. The accession numbers of the sequences used are listed in the parentheses.

**Figure 2:**
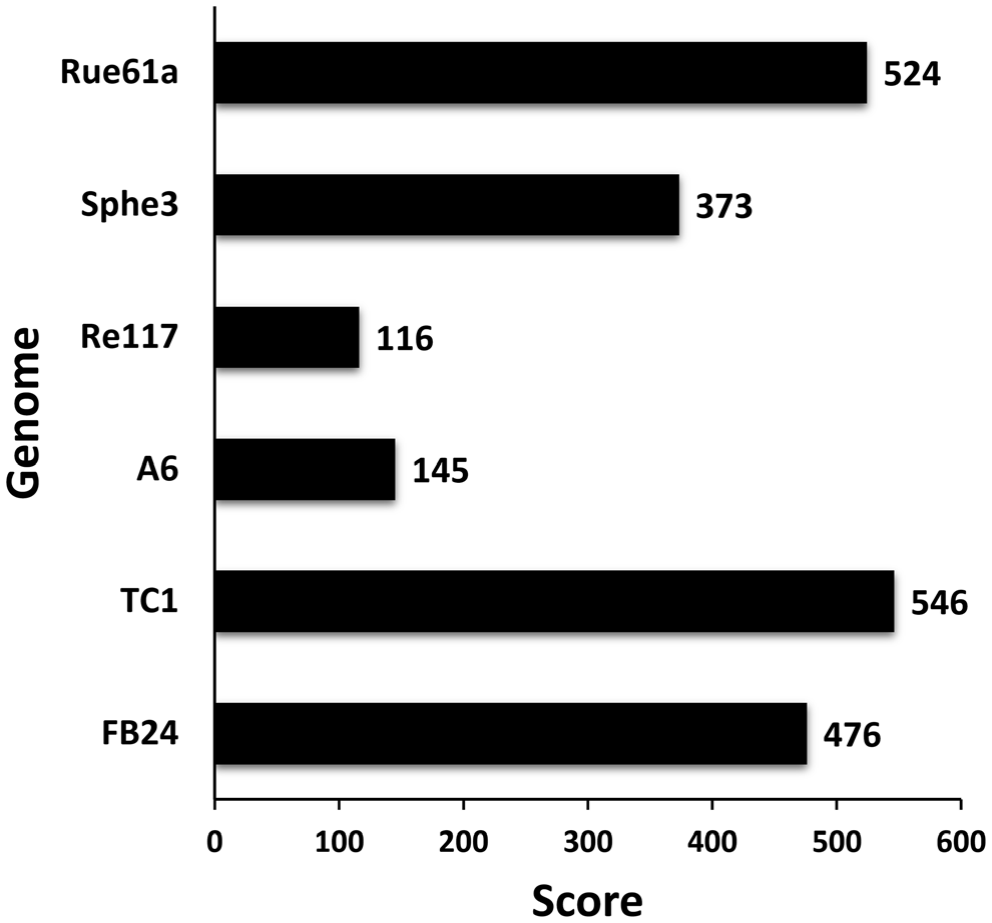
Genome similarity analysis of strain M2012083. The genomes of the seven *Arthrobacter* strains were submitted to the web service RAST for comparison. The genome of M2012083 was compared with the other six complete genomes and the comparison scores were shown. Higher score means higher similarity.

The nicotine-degrading strain M2012083 was isolated from tobacco waste and deposited in the China Center for Type Culture Collection (CCTCC; collection number M2012083). Strain M2012083 is gram-positive, aerobic, and asporous. Transmission electron microscopic observation revealed that the cells of strain M2012083 were long rods in their lag phase (6 h) and became coccus-shaped during the log phase (36 h) in nicotine-containing medium. These morphological characteristics are representative of the genus *Arthrobacter*, but were also detected in members of other genera such as *Brevibacterium* and *Rhodococcus*^50^. The results of carbon source oxidation tests, enzyme activity tests, and carbon source utilization tests of strain M2012083 are shown in Table S1, Table S2, and Table S3, respectively. The 16S rDNA sequence of strain M2012083 was sequenced and submitted to GenBank (accession number KF893303). Nucleotide BLAST searches of the GenBank database and Ribosomal Database Project library (http://blast.ncbi.nlm.nih.gov/Blast.cgi) indicated that the 16S rDNA sequence of strain M2012083 exhibited more than 99% nucleotide acid sequence identities with that of TC1 and Rue61a. We selected 55 16S rDNA sequences from different strains in order to construct a phylogenetic tree by using MEAG5 analysis with the neighbor-joining method^51^,^52^; strain M2012083 is closest in phylogenetic tree to type strains of *A.* *aurescens* and *A.* *nitroguajacolicus* (Fig. 1). Furthermore, genome comparison using the RAST Prokaryotic Genome Annotation Server showed that the genomic sequence of M2012083 was more similar to the complete genome of TC1 (comparison score: 546) than to that of Rue61a (comparison score: 524) (Fig. 2). Based on the above results, strain M2012083 was classified as *A. aurescens.*

### General genome features and comparative genomics

**Figure 3:**
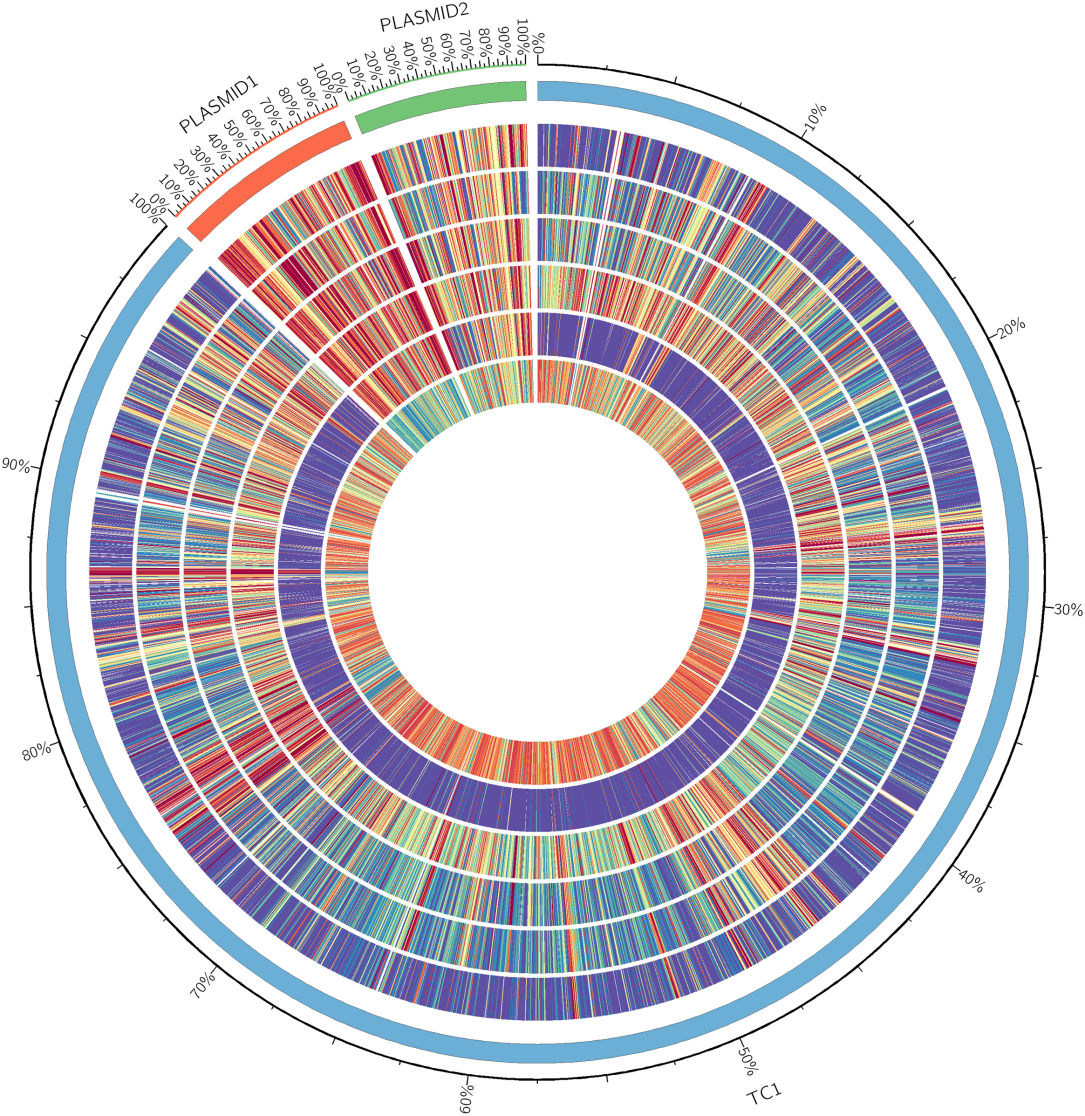
Comparison of the genome of *Arthrobacter aurescens* M2012083 with other *Arthrobacter* genomes. The outermost circle (circle 1) represents the scale. Circle 2, the chromosomal (blue) and plasmids (orange and green) open reading frames (ORFs) of *A. aurescens* TC1 as references; circle 3, ORFs of *A. chlorophenolicus* A6 complete genome; circle 4, ORFs of *A.* *nitroguajacolicus* Rue61a complete genome; circle 5, ORFs of *A. arilaitensis* Re117 complete genome; circle 6, ORFs of *A. phenanthrenivorans* Sphe3 complete genome; circle 7, ORFs of *A.* sp. FB24 complete genome; circle 8, ORFs of *A. aurescens* M2012083 complete genome. ORFs are represented by colorful sticks (red-to-blue were assigned according to the similarity of the ORF to the homolog in TC1 genome) in circles 3 to 8.

**Figure 4:**
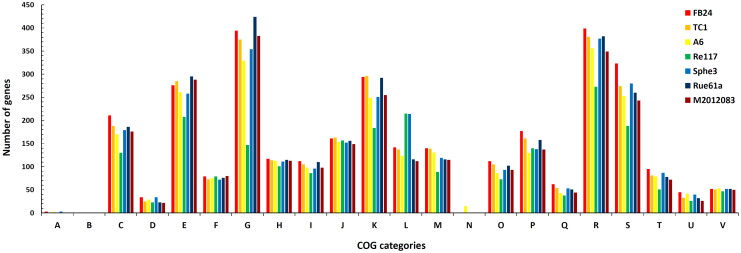
Comparison of COG categories among seven subject *Arthrobacter* strains. Functional comparisons among the genomes of *Arthrobacter* sp. FB24, *A.* *aurescens* TC1, *A. chlorophenolicus* A6, *A. arilaitensis* Re117, *A. phenanthrenivorans* Sphe3, *A.* *nitroguajacolicus* Rue61a and *A. aurescens* M2012083 were performed based on the functional classifications of COG database^54^. The ordinate axis represents the number of genes in each COG functional category. The 22 COGs categories are as following: RNA processing and modification (A); chromatin structure and dynamics (B); energy production and conversion (C); cell cycle control, cell division, chromosome partitioning (D); amino acid transport and metabolism (E); nucleotide transport and metabolism (F); carbohydrate transport and metabolism (G); coenzyme transport and metabolism (H); lipid transport and metabolism (I); translation, ribosomal structure and biogenesis (J); transcription (K); replication, recombination and repair (L); cell wall, membrane, envelope biogenesis (M); cell motility (N); posttranslational modification, protein turnover, chaperones (O); inorganic transport and metabolism (P); secondary metabolites biosynthesis, transport and catabolism (Q); general function prediction only (R); function unknown (S); signal transduction mechanisms (T); intracellular trafficking, secretion and vesicular transport (U); defense mechanisms (V).

The general features of the seven subject *Arthrobacter* genomes are summarized in Table 1. Pan-genome analyses, such as an overview of genomic conserved regions, orthologous group categorization, and orthologous relationship analysis, were performed in order to identify conserved and strain-specific CDSs. BLASTatlas tool was used to compare the genomes of the seven *Arthrobacter* strains and to provide a quick overview of the conserved genomic regions^53^ (Fig. 3). The genome of TC1 was used as a reference because it is most similar to the genome of M2012083 and has been thoroughly studied^18^,^27^ (Fig. 1, 2). For the seven different *Arthrobacter* genomes, 68–79% of protein-coding genes were functionally categorized based on the Clusters of Orthologous Groups (COGs) database^54^ (Fig. 4).

**Figure 5:**
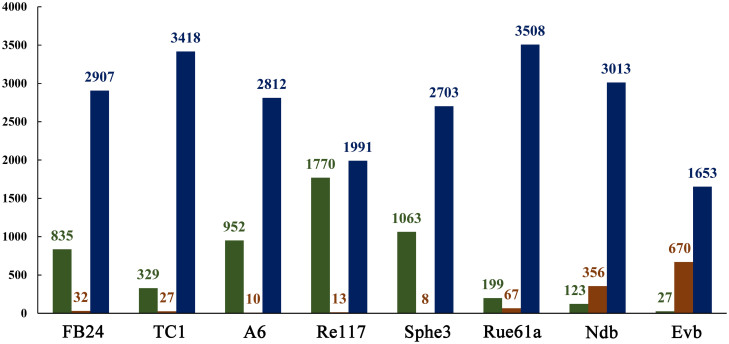
Numbers of conversed and specific groups in M2012083 compared with six other *Arthrobacter* genomes. This analysis is based on the results of orthologous relationships analysis which is shown in DataSet1. Green rectangle, orthologous groups specific in M2012083 compared with the strain(s) of the horizontal axis; brown rectangle, orthologous groups conserved in M2012083 compared with the strain(s) of the horizontal axis but were not found in other studied strain(s); blue rectangle, orthologous groups conserved in M2012083 compared with the strain(s) of the horizontal axis and also were found in some other studied strain(s); studied strains, strains M2012083, FB24, TC1, A6, Re117, Sphe3 and Rue61a; Ndb, *N*-heterocyclic compounds degradation bacteria TC1 and Rue61a; Evb, environmental bacteria FB24, TC1, A6, Sphe3 and Rue61a.

Orthologous relationship analysis was performed using the OrthoMCL method^55^. All protein-coding genes of the seven subject strains were clustered into a total of 5,368 orthologous groups with 1,653 conserved groups, while the protein-coding genes of M2012083 were clustered into a total of 3,774 groups (DataSet1, Fig. 5). The strain-specific CDSs of each of the seven *Arthrobacter* strains, which have no homologous genes in the other six strains, are listed in DataSet2, along with their ORF IDs and annotations.

### Nicotine catabolism

**Figure 6:**
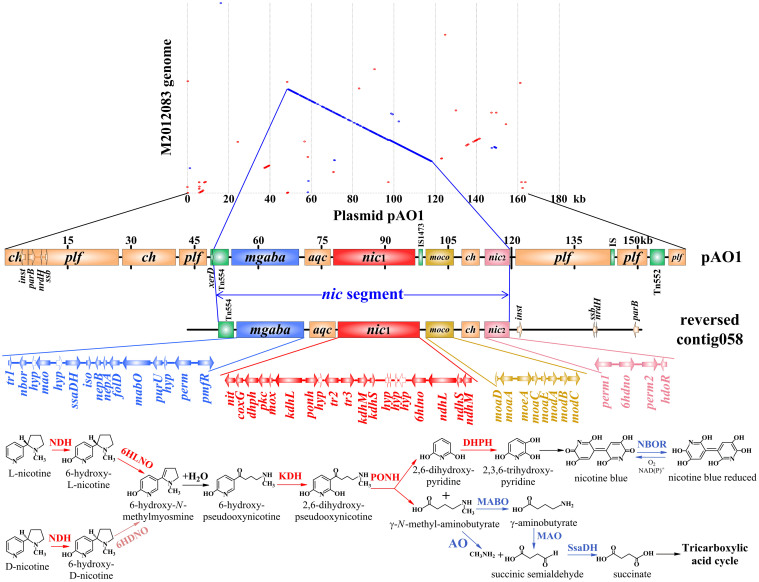
Comparison among the genome of M2012083 and plasmid pAO1. The synteny plot of plasmid pAO1 versus M2012083 genome was generated by using MUMmer program. Red ringlets indicate the forward conserved genes between M2012083 genome and plasmid pAO1; blue ringlets indicate the reverse conserved genes between M2012083 genome and plasmid pAO1. The names, arrangements and functions of genes, and the nicotine-degrading pathway in *Arthrobacter* are shown according to Ganas P, Igloi GL and Brandsch R^38^. The rectangles in different colors represent gene clusters involved in carbohydrate catabolism (*ch*), plasmid function (*plf*), γ-*N*-methylaminobutyrate catabolism (*mgaba*), assembly and quality control of the (αβγ)_2_ holoenzyme complexes of NDH and KDH (*aqc*), nicotine degradation (*nic1* and *nic2*), molybdenum cofactor biosynthesis (*moco*) and transposons (Tn554), and some insertion sequences (IS and IS1473). ORFs were indicated by arrows, and the hollow arrows represent hypothetical protein genes (*hyp*). See text and DataSet3 for details.

In order to reveal the nicotine-degradation mechanism of strain M2012083, its genome was searched for all reported bacterial nicotine degradation-related genes and the protein-coding genes of plasmid pAO1, using the tBLASTp program^37^,^38^,^41^,^42^,^43^,^56^,^57^,^58^,^59^. In the M2012083 genome, there are homologs of 94 of the total 166 ORFs of plasmid pAO1, including all 52 nicotine degradation-related ORFs and some carbohydrate catabolism-related ORFs (Fig. 6, DataSet3).

### Survival capacity of *Arthrobacter* strains

Genome analysis and comparisons showed that more than 100 ORFs related to survival capacity occurred in all subject *Arthrobacter* strains except Re117, which contained only 74 ORFs (Table 2). ORFs involved in survival capacity are listed in detail for all subject *Arthrobacter* strains in DataSet4.

## Discussion

The assembled genome of M2012083 had approximately 329-fold sequence coverage^49^, with 4,312 protein-coding genes. The genome size of the seven subject *Arthrobacter* strains ranges from 3.8 to 5.3 Mbp. The smallest genome is that of Re117, which is the only food-processing bacterium among the subject strains, resulting in the fewest protein-coding genes (Table 1); this suggests that living on the surface of cheese requires the fewest genes. To a certain extent, the different genomic G + C content of the seven studied *Arthrobacter* strains can provide data on the links between G + C content, genetic relationship, and function. For example, *N*-heterocyclic pollutant-degrading strains M2012083, TC1 and Rue61a, which are close on the phylogenetic tree, show similar G + C content, while strains A6 and Re117, which are far away from M2012083 on the phylogenetic tree, show the highest and the lowest G + C content, respectively (Table 1, Fig. 1). In the genome of M2012083, there are 1,694 conserved CDSs, most of which are related to cell cycle control, translation, and nucleotide transport and metabolism; conserved CDSs involved in carbohydrate transport and metabolism are the fewest, indicating that the kinds of carbohydrate that the seven subject *Arthrobacter* strains utilize are very different (Table 1, Fig. 3, DataSet1). In addition, there are 434 strain-specific CDSs in M2012083, including most of the nicotine-degrading genes (Table 1, Fig. 3, DataSet2). COG comparisons among the seven *Arthrobacter* genomes showed that the number of genes involved in carbohydrate transport and metabolism (COG G) was highest in the genomes of the six environmental *Arthrobacter* strains, FB24, TC1, A6, Sphe3, Rue61a, and M2012083, while genes related to replication, recombination, and repair (COG L) were the most numerous in the Re117 genome, in which the percentage of COG G proteins (6.5%) was much lower than that in the other subject genomes (>11.8%); this is probably because strain Re117 locates on the surface of cheese, which is rich in proteins rather than carbohydrates. Furthermore, the numbers of genes categorized as COG C, K, R, S, and T in the Re117 genome are also much lower than those in other subject genomes, which indicates that surviving on the surface of cheese requires less functions related to energy transformation, transcription, or signal transduction. The COGs have highly similar distributions among the six environmental *Arthrobacter* strains, except for COGs L and N. Genes with COG L annotations, representing replication, recombination, and repair, are more numerous in Sphe3 than in the other five strains. There are 15 genes annotated as COG N in A6, which is far more than that in other strains (only 0 or 1), suggesting that all the subject *Arthrobacter* strains lacked cell motility, except strain A6. Genes annotated as COG B, the group related to chromatin structure and dynamics, were not found in any of the seven subject genomes (Fig. 4). Comparisons between the M2012083 genome and each of the other six subject genomes indicated that 3,575 orthologous groups in M2012083 were conserved in Rue61a, while 199 groups were specific to M2012083 compared with Rue61a. In contrast, M2012083 and Re117 only share 2,004 conserved groups, and 1,770 groups in M2012083 are specific to M2012083 compared with Re117. There were 3,369 conserved groups among the genomes of the *N*-heterocyclic pollutant-degrading bacteria, M2012083, TC1, and Rue61a, including 356 groups that were not found in the other four subject genomes. Among the six environmental *Arthrobacter* strains, M2012083, FB24, TC1, A6, Sphe3, and Rue61a, 2,323 conserved groups were identified, including 670 groups that were not found in the food-processing strain, Re117 (Fig. 5).

All the nicotine degradation-related genes, such as those encoding nicotine dehydrogenase subunits (*ndhL*, *ndhM*, and *ndhS*), 6-hydroxy-L-nicotine oxidase (*6hlno*), 6-hydroxy-D-nicotine oxidase (*6hdno*), and ketone dehydrogenase subunits (*kdhL*, *kdhM*, and *kdhS*), and those involved in γ-*N*-methylaminobutyrate catabolism (*mgaba*), MoCo biosynthesis (*moco*), assembly and quality control of the (αβγ)_2_ holoenzyme complexes (*aqc*), compound transport (*perm1* and *perm2*), and gene regulation (*tr1*, *tr2*, and *tr3*), were located on a 68,622-bp DNA segment (*nic* segment-1) of contig 058 in the M2012083 genome, which showed 98.1% nucleotide acid sequence identity to the 69,252-bp *nic* segment of pAO1 (Fig. 6, DataSet3). Furthermore, the only nicotine degradation-related ORFs absent from plasmid pAO1, the two which encode the MoeB protein and a MogA homolog that are essential for MoCo biosynthesis were also identified in the M2012083 genome (1197706.3.peg.1697 and 1197706.3.peg.1089). However, the *xerD* integrase gene and the IS1473 insertion sequence that contains the resolvase gene are absent from the *nic* segment-1 of M2012083, while the sequence of the Tn554 transposon is incomplete within it (Fig. 6, DataSet3). Plasmid pAO1 sequences other than the *nic* segment, which contain all the plasmid-function genes, show no significant similarity to the M2012083 genome sequences (Fig. 6). Most of the pAO1 ORFs involved in plasmid function, such as the ORFs involved in replication (CDS_28 and CDS_120), partitioning (PAR_1 and CDS_9), maintenance (CDS_21 and CDS_22), conjugation (CDS_5, CDS_6, CDS_83, and CDS_92), stress response (CDS_7), abortive infection phage resistance (CDS_118), cell-cell contact (CDS_18), and cell signaling (CDS_113), and many predicted membrane proteins that may participate in conjugation (CDS_8, CDS_14, CDS_30, CDS_31, CDS_84, CDS_85, CDS_86, CDS_87, CDS_88, CDS_89, CDS_90, CDS_91, and CDS_114), were not found in the M2012083 genome (DataSet3)^37^,^38^. These data suggest that the nicotine degradation-related genes of *A*. *aurescens* M2012083 are located on the chromosome or a plasmid other than pAO1.

It has been reported that alternative σ-factor induction is an important strategy for bacteria to deal with environmental stress^27^. The genome of M2012083 encodes 35 transcription factors; these include 17 members of the σ^70^ family of σ factors, this number is similar to that in the other two *N*-heterocyclic pollutant-degrading *Arthrobacter* strains (TC1 and Rue61a) and considerably more than that in strain Re117, which only encodes six (Table 2). The expression of universal stress-related proteins (USPs) is induced in cells in response to heat shock, oxidant and UV exposure, carbon, nitrogen, and phosphate starvation, and entering stationary phase^60^. As in strain TC1, M2012083 contains eight potential USPs, far fewer than FB24 and Rue61a (15 and 13, respectively) but more than Re117 (Table 2, DataSet4). In addition, ORFs involved in osmotic and oxidative stress responses, cold- and heat-shock responses, detoxification, and carbon starvation are present, and distributed differently, among strain M2012083 and the other subject *Arthrobacter* strains (Table 2, DataSet4).

Trehalose, glycogen, and glycine betaine can help bacteria tolerate osmotic stress^61^,^62^,^63^. Many ORFs associated with the synthesis and catabolism of trehalose and glycogen were identified in the six environmental *Arthrobacter* strains, while no trehalose synthase or glycogen debranching ORFs were identified in strain Re117. These findings suggest that all the subject strains, except Re117, can synthesize or catabolize trehalose and glycogen in order to regulate intracellular osmotic pressure when necessary. Although they have different choline-intake abilities, all seven *Arthrobacter* strains can use exogenic choline as a substrate for synthesizing betaine in order to maintain osmotic balance. An operon that includes the choline dehydrogenase gene (*betA*) and betaine aldehyde dehydrogenase gene (*betB*), and a gene cluster that encodes the four subunits of sarcosine oxidase, are present in all seven *Arthrobacter* genomes. A second copy of *betA* was identified in FB24, TC1, and A6, while a second copy of *betB* is found only in FB24 and A6. Many glycine betaine transporter genes, such as clusters of the glycine betaine ABC transport system permease and *opuD* genes, were also present in all seven *Arthrobacter* genomes. Only strains FB24 and A6 contained both the choline binding protein A gene and high-affinity choline uptake protein (BetT) gene; only strains M2012083, TC1, Sphe3, and Rue61a contained the choline binding protein A gene, while only strain Re117 contained the *betT* gene. Moreover, in strain M2012083, osmotic stress may be reduced by the regulation of water movement, glycerol uptake, and osmoregulated periplasmic glucan synthesis, via aquaporin Z, the glycerol uptake facilitator protein, and opgC, respectively (DataSet4).

About 30 ORFs that respond to oxidative stress were identified in every subject *Arthrobacter* genome (Table 2, DataSet4). Most of the ORFs, such as those for catalases (EC 1.11.1.6), ferroxidases (EC 1.16.3.1), and superoxide dismutases (EC 1.15.1.1), were conserved among the seven genomes, while a peroxidase (EC 1.11.1.7), which was conserved in A6, Re117, and Sphe3, was not found in M2012083, FB24, TC1, or Rue61a. In addition, all the subject *Arthrobacter* genomes encode glutathione peroxidase (EC 1.11.1.9), except that of the food-processing strain, Re117. An ORF encoding SoxR, which is a redox-sensitive activator of the oxidative stress response, is present in M2012083, TC1, Re117, and Rue61a but absent from FB24, A6, and Sphe3 (DataSet4). These data suggest that induced SoxR directly regulates the expression of oxidative stress response genes in strains M2012083, TC1, Re117, and Rue61a, while strains FB24, A6, and Sphe3 do not have this regulation mechanism. No SoxS ORF was found in any subject *Arthrobacter* strains, as in many other environmental bacteria^64^.

Three to five cold-shock protein *cspA* genes were found in environmental *Arthrobacter* strains (M2012083, FB24, TC1, A6, Sphe3, and Rue61a), while only one was found in the food-processing *Arthrobacter* strain, Re117. Every subject genome contained an ORF encoding cold-shock protein CspC, except the genome of Rue61a. In addition, all the *Arthrobacter* strains contained a *dnaK* operon (encoding chaperone proteins DnaJ and DnaK, and heat shock protein GrpE), an *hspR* gene encoding a transcriptional repressor of the *dnaK* operon, a heat-inducible transcription repressor *hrcA* gene, and several other genes encoding heat-shock response proteins. Interestingly, an additional one or two *dnaJ*, *dnak*, and *grpE* genes were identified in the M2012083 genome, but these genes were not located in an operon; similar data were observed in strain Rue61a (DataSet4).

Selenium is essential for living organisms, at low concentration, and occurs extensively in the Earth's crust. However, in aerobic environments, selenium occurs as selenate and selenite, which are toxic and mutagenic at excess concentration. The *dedA* gene is reported to be very important for the regulation of selenite uptake in a high selenite-resistant bacterium, *Ralstonia metallidurans* CH34^65^. Three *dedA* genes were identified in strain M2012083 and two in FB24, TC1, A6, Sphe3, and Rue61a, while only one *dedA* gene was identified in Re117. However, only the ATP-binding component of the selenite uptake transporter is present in all the subject strains, while the periplasmic substrate-binding and permease components of the ABC transporter exist only in strains A6 and Sphe3 (DataSet4). D-Amino acids may be toxic to growing cells because they may be mistakenly attached to tRNAs. D-Tyrosyl-tRNA^Tyr^ deacylase can remove misattached D-amino acids from tRNAs, thus detoxifying D-amino acids^66^. A Re117-specific D-tyrosyl-tRNA^Tyr^ deacylase ORF was detected, which suggests that Re117 is capable of detoxifying D-amino acids, while other subject *Arthrobacter* strains are not (DataSet4).

In addition to σ^70^ factors, σ^B^ and RpoS family members, which are alternative σ factors for RNA polymerase transcription, play major roles in the general stress response and environmental survival of bacteria. σ^B^ activity is inhibited by anti-σ^B^ factor RsbW, while it is stimulated by serine phosphatase RsbU and anti-σ^B^ factor antagonist RsbV^67^. Strains M2012083, FB24, TC1, A6, and Rue61a contain genes that encode RNA polymerase σ factor SigB, RsbU, and RsbV, while strain Sphe3 contains genes that encode SigB and RsbU. In contrast to other subject *Arthrobacter* strains, strain Re117 does not contain any genes involved in σ^B^ stress-response regulation. The *rsbW* gene was not detected in any of the subject strains (DataSet4).

During the response to carbon starvation, *rpoS* gene expression is inhibited by RpsA, which is a starvation-sensing protein^68^. The *rpsA* gene and a carbon starvation protein A gene were identified in strain M2012083 and the other environmental subject strains. In contrast, genes involved in the carbon starvation response were not detected in strain Re117 (DataSet4). Expression of the *rpoS* gene is also regulated by homoserine lactones (HSLs) and a derivative thereof^68^. Genes encoding aspartate kinase, aspartate semialdehyde dehydrogenase, and homoserine dehydrogenase, which has been suggested catalyze HSL synthesis from L-asparate^27^, were identified in the M2012083 genome (1197706.3.peg.3479, 1197706.3.peg.1452, and 1197706.3.peg.2912, respectively) and the other six *Arthrobacter* genomes (DataSet1). Based on all the genome analyses, strain Re117 contains the fewest carbohydrate metabolism and stress-response genes of the seven subject *Arthrobacter* strains; this is probably because the habitat of Re117, the surface of cheese, is much more stable than soil.

## Methods

### Strain identification

The M2012083 strain was cultured at 30°C and 220 rpm in lysogenic broth or nicotine medium, as previously described^69^. Morphological characteristics were observed using a transmission electron microscope. Physiological and biochemical properties, such as the oxidation and utilization of different carbon sources, and the activities of different enzymes were determined by the CCTCC.

The 16S rDNA of strain M2012083 was amplified by PCR with the universal primer pair of 27F (5′-AGAGTTTGATCCTGGCTCA-3′) and 1492R (5′-GGTTACCTTGTTACGACTT-3′) from extracted genomic DNA. The PCR product was purified and sequenced, then analyzed by homology alignment using the BLAST search program (http://www.ncbi.nlm.nih.gov/BLAST.html). A phylogenetic tree was constructed by the neighbor-joining method using MEGA5 analysis^51^,^70^.

### Genome sequencing and annotation

The genomic DNA of M2012083 was extracted using the Wizard Genomic DNA Purification Kit (Promega, Madison, WI, USA). Whole-genomes of strain M2012083 were sequenced by the Chinese National Human Genome Center (Shanghai, China). The Velvet program was used to assemble the pair-end reads *de novo* with manually optimized settings. The genome sequences of FB24, TC1, A6, Re117, Sphe3, and Rue61a were obtained from GenBank. The genomes of all these *Arthrobacter* strains were submitted to the RAST web service for automated annotation followed by manual checking. The annotations are available via a guest account at the RAST website. Information on COGs and from the Conserved Domain Database was also used for comparisons.

### Genome comparison and analysis

The National Institutes of Allergy and Infectious Diseases/Pathosystems Resource Integration Center (PATRIC) and RAST were used for genome comparisons and metabolic pathway analyses. The tBLASTn program was used to search for homologs of plasmid pAO1 protein-coding genes in the M2012083 genome with coding amino acid sequence identity > 30% at an e-value < 1e-5. The MUMmer program was used to generate a synteny plot of plasmid pAO1 versus the M2012083 genome. The OrthoMCL method was used to analyze the orthologous relationships between protein-coding genes in different genomes. Genes in different genomes with coding amino acid sequence identity > 50% at an e-value < 1e-5 were identified as orthologous genes. We used the CRISPRFinder program in CRISPR's web server to identify clustered regularly interspaced short palindromic repeats in the CRISPR/Cas RNA-guided nuclease system^71^. The genomic context was visualized using the Circos software.

### Accession numbers

The genome sequences of *Arthrobacter aurescens* M2012083 has been deposited in NCBI database under the accession number AKKK00000000. The accession numbers of the subject genomes are as following: *Arthrobacter* sp. FB24 chromosome, NC_008541; *Arthrobacter* sp. FB24 plasmid 1, NC_008537; *Arthrobacter* sp. FB24 plasmid 2, NC_008538; *Arthrobacter* sp. FB24 plasmid 3, NC_008539; *Arthrobacter aurescens* TC1 chromosome, NC_008711; *Arthrobacter aurescens* TC1 plasmid TC1, NC_008712; *Arthrobacter aurescens* TC1 plasmid TC2, NC_008713; *Arthrobacter chlorophenolicus* A6 chromosome, NC_011886; *Arthrobacter chlorophenolicus* A6 plasmid pACHL01, NC_011879; *Arthrobacter chlorophenolicus* A6 plasmid pACHL02, NC_011881; *Arthrobacter arilaitensis* Re117 chromosome, NC_014550; *Arthrobacter arilaitensis* Re117 plasmid pRE117-1, NC_014549; *Arthrobacter arilaitensis* Re117 plasmid pRE117-2, NC_014548; *Arthrobacter phenanthrenivorans* Shpe3 chromosome, NC_015145; *Arthrobacter phenanthrenivorans* Shpe3 plasmid pASPHE301, NC_015146; *Arthrobacter phenanthrenivorans* Shpe3 plasmid pASPHE302, NC_015147; *Arthrobacter nitroguajacolicus* Rue61a chromosome, NC_018531; *Arthrobacter nitroguajacolicus* Rue61a plasmid pAL1, NC_009453; *Arthrobacter nitroguajacolicus* Rue61a plasmid p232, NC_018532; *Arthrobacter nicotinovorans* plasmid pAO1, NC_021229.

## Author Contributions

P.X., Y.Y. and H.T. conceived and designed the project and experiments. Y.Y., S.F. and H.T. performed the computational and bioinformatic analysis. Y.Y., H.T. and P.X. wrote the paper. All authors reviewed the paper.

## Supplementary Material

Supplementary Information Supplementary information

Supplementary Information Dataset 1

Supplementary Information Dataset 2

Supplementary Information Dataset 3

Supplementary Information Dataset 4

## Figures and Tables

**Table 1:** General genome features of the seven subject *Arthrobacter* strains

Genome feature	*Arthrobacter* strain						
	M2012083	FB24	TC1	A6	Re117	Sphe3	Rue61a
Size (base pair)	4,629,172	5,070,478	5,226,648	4,980,870	3,918,192	4,535,320	5,081,038
G + C content	62.0%	65.4%	62.4%	66.0%	59.3%	65.4%	62.2%
Protein-coding genes	4312	4523	4588	4590	3436	4131	4575
No. of protein-coding genes with function prediction	3254 (75.5%)	3256 (72.0%)	3366 (73.4%)	3095 (67.4%)	2378 (69.2%)	2922 (70.7%)	3168 (69.2%)
No. of protein-coding genes without function prediction	1058 (24.5%)	1267 (28.0%)	1222 (26.6%)	1495 (32.6%)	1058 (30.8%)	1209 (29.3%)	1407 (30.8%)
No. of protein-coding genes with COGs	3114 (72.2%)	3567 (78.9%)	3361 (73.3%)	3125 (68.1%)	2473 (72.0%)	3264 (79.0%)	3367 (73.6%)
RNA genes	58	69	73	104	82	65	71
rRNA genes(5S rRNA, 16S rRNA, 23S rRNA)	4(2, 1, 1)	15(5, 5, 5)	18(6, 6, 6)	15(5, 5, 5)	18(6, 6, 6)	12(4, 4, 4)	18(6, 6, 6)
tRNA genes	54	51	54	88	64	50	53
Other RNA genes	0	3	1	1	0	3	0
Conserved CDS^a^	1694	1717	1722	1703	1677	1688	1704
Strain-specific CDS^b^	434	540	427	788	816	417	328

The orthologous relationship analysis was performed by OrthoMCL.

^a^: Protein-coding genes having orthologous genes in every other subject genomes;

^b^: Protein-coding genes not having orthologous gene in any other subject genomes.

**Table 2:** Comparison of the number of ORFs related to survival capacity among the seven subject *Arthrobacter* genomes

*Arthrobacter* strain	Number of ORFs related to:								
	Transcription factors	σ^70^ factors	USPs	Osmotic stress	Oxidative stress	Cold and heat shock	Detoxification	Other stress	Total^a^
M2012083	35	17	8	35	29	21	4	12	109
FB24	29	13	15	33	31	18	3	11	111
TC1	34	17	8	32	30	19	3	12	104
A6	28	12	11	34	30	20	6	11	112
Re117	22	6	6	20	26	15	3	4	74
Sphe3	27	10	11	30	32	19	6	10	108
Rue61a	35	18	13	35	31	20	3	12	114

^a^: Total number of ORFs related to UPSs, osmotic stress, oxidative stress, cold and heat shock, detoxification and other stress.
