# Supplementary material for: Comparative genome analysis reveals the molecular basis of nicotine degradation and survival capacities of Arthrobacter
Source: Sci Rep. 2015 Feb 27;5:8642. doi: 10.1038/srep08642 (PMC4342571; doi:10.1038/srep08642)
Supplement: Supplementary Information [file srep08642-s1.pdf]

Supplementary information

Comparative genome analysis reveals the molecular basis of nicotine degradation and survival capacities of *Arthrobacter*

Yuxiang Yao[1, 2†], Hongzhi Tang[1, 2†], Fei Su[1, 2] and Ping Xu[1, 2#]

[1]State Key Laboratory of Microbial Metabolism, and School of Life Sciences & Biotechnology, Shanghai Jiao Tong University, Shanghai 200240, People's Republic of China

[2]Joint International Research Laboratory of Metabolic & Developmental Sciences, Shanghai Jiao Tong University, Shanghai 200240, People's Republic of China

[*]Running title: Comparative genome analysis of *Arthrobacter*

[#]Address correspondence to: Ping Xu, School of Life Sciences and Biotechnology, Shanghai Jiao Tong University, Shanghai 200240, PR China. Tel: +86-21-34206723; Fax: +86-21-34206723; E-mail: pingxu@sjtu.edu.cn

[†]These authors contributed equally to this work.

Table S1

Oxidation of carbon sources by strain M2012083.

Test (oxidation of carbon sources)		Result	Test (oxidation of carbon sources)		Result
0	Control	-	25	Polychrom	+
1	Glycerol	+	26	Saligenin	-
2	Erythritol	+	27	Cellobiose	+
3	D-Arabinose	-	28	Maltose	-
4	L-Arabinose	-	29	Lactose	-
5	Ribose	-	30	Melibiose	W
6	D-Xylose	W	31	Sucrose	-
7	L-Xylose	-	32	Trehalose	-
8	Adonitol	-	33	Synanthrin	-
9	β-Methyl-D-xyloside	W	34	Melezitose	-
10	Galactose	-	35	Raffinose	-
11	Glucose	+	36	Starch	-
12	Fructose	+	37	Glycogen	-
13	Seminose	-	38	Xylitol	-
14	Sorbose	-	39	Gentiobiose	-
15	Rhamnose	-	40	D-Turanose	+
16	Dulcitol	-	41	D-Lyxose	-
17	Inositol	+	42	D-Tagatose	-
18	Mannitol	-	43	D-Fucose	-
19	Sorbitol	-	44	L-Fucose	-
20	α-Methyl-D-mannoside	-	45	D-Arabitol	-
21	α-Methyl-D-glucoside	-	46	L-Arabitol	-
22	*N*-Acetyl-glucosamine	-	47	Gluconate	-
23	Amygdalin	+	48	2-Keto-gluconate	-
24	Arbutin	-	49	5-Keto-gluconate	-

-, negative reaction; +, positive reaction; W, weak positive reaction.

Table S2

Enzyme activities of strain M2012083.

Enzyme	Result	Enzyme	Result
Alkaline phosphatase	+	α-Galactosidase	+
Esterase (C4)	W	β-Galactosidase	+
Lipoid esterase (C8)	+	β-Glucuronidase	+
Lipase (C14)	-	α-Glucosaccharase	+
Leucine aromatic aminopeptidase	+	β-Glucosaccharase	+
Valine aromatic aminopeptidase	-	*N*-Acetyl-glucosaminidase	+
Cystine aromatic aminopeptidase	W	α-Mannosidase	+
Trypsin	-	β-Fucosidase	+
Chymotrypsin	-	Arginine dihydrogenase	-
Acid phosphatase	+	Urease	-
Naphthol-AS-BI-phosphohydrolase	W	Gelatinase	+
Nitrate reduction	-	Indole production	-

-, negative reaction; +, positive reaction; W, weak positive reaction.

Table S3

Utilization of carbon sources by strain M2012083.

Test<br>(utilization of carbon sources)	Result	Test<br>(utilization of carbon sources)	Result
Water	-	D-Tagatose	-
α-Cyclodextrin	-	D-Trehalose	-
β-Cyclodextrin	-	Turanose	+
Dextrin	+	Xylitol	-
Glycogen	W	D-Xylose	W
Inulin	-	Acetate	+
Mannosan	-	α-Hydroxybutyrate	+
Tween-40	+	β-Hydroxybutyrate	+
Tween-80	+	γ-Hydroxybutyrate	+
N-Acetyl-D-galactosamine	+	*p*-Hydroxyphenylacetic acid	+
N-Acetyl-D-glucosamine	-	α-Ketoglutarate	+
Amygdalin	+	α-Oxopentanoic acid	+
L-Arabinose	+	Lactamide	+
D-Arabinose	-	D-Methyl lactate	-
Arbutin	+	L-Lactate	+
D-Cellobiose	+	D-Malate	-
D-Fructose	+	L-Malate	W
L-Fucose	+	Methyl pyruvate	+
D-Galactose	+	mono-Methyl succinate	+
D-Galacturonic acid	-	Propionic acid	+
Gentiobiose	+	Pyruvate	+
D-Gluconic acid	+	Succinamic acid	+
α-D-Glucose	+	Succinate	-
m-Inositol	+	*N*-Acetyl-L-glutamate	+
α-D-Lactose	+	L-Alaninamide	+
Lactulose	+	D-Alanine	+
Maltose	+	L-Alanine	+
Maltotriose	+	L-Alanyl-glycine	+
D-Mannitol	+	L-Asparagine	+
D-Mannose	+	L-Glutamate	+
D-Melezitose	+	Glycyl-L-glutamine	+
D-Melibiose	+	L-Pyroglutamic acid	+
α-Methyl-D-galactoside	+	L-Serine	+
β-Methyl-D-galactoside	+	Butanediamine	+
3-Methyl-D-glucose	-	2,3-Butanediol	-
α-Methyl-D-glucoside	-	Glycerol	+

β-Methyl-D-glucoside	-	Adenosine	+
α-Methyl-D-mannoside	-	2'-Deoxyadenosine	-
6-*O*-D-Glucopyranosyl-D-fructo-furanose	+	Inosine	+
D-Allulose	+	Thymidine	-
D-Melitriose	+	Uridine	-
L-Rhamnose	+	5'-Adenosine monophosphate	+
D-Ribose	+	5'-Thymidine monophosphate	-
Saligenin	+	5'-Uridine monophosphate	+
Sedoheptulosan	-	6-Phosphate-D-fructose	-
D-Sorbitol	+	1-Phosphate-α-D-glucose	-
Stachyose	+	6-Phosphate-D-glucose	-
Sucrose	+	D-L-α-Phosphoglycerol	+
Decanoate	-	Adipic acid	-
Citrate	+	Phenylacetic acid	W

-, negative reaction; +, positive reaction; W, weak positive reaction.
